# A Rare Case Report and Literature Review of External Auditory Canal Cholesteatoma with Circumferential Destruction of Canal Wall Exposing Facial Nerve

**DOI:** 10.1155/2017/7450482

**Published:** 2017-12-28

**Authors:** Leison Maharjan, Pabina Rayamajhi

**Affiliations:** Department of ENT-HNS, Institute of Medicine (IOM), Kathmandu, Nepal

## Abstract

External auditory canal cholesteatoma (EACC) is a rare condition with an estimated incidence of 1.2 per 1000 new otological patients. It is often mistaken with keratosis obturans. We discuss an extensive primary EACC with an aural polyp in a male which was managed by modified radical mastoidectomy.

## 1. Introduction

External auditory canal cholesteatoma (EACC) is a rare condition with an estimated incidence of 1.2 per 1000 new otological patients [1]. Compared to the incidence rate of middle ear cholesteatoma which is 9.2 per year per 100,000, the incidence of primary EACC is 0.30 per 100,000 population [2, 3]. EACC is often mistaken with keratosis obturans. Rarely such cases with circumferential EAC involvement have been reported with an additional exposure of the facial nerve. We discuss a clinical, radiological, and preoperative finding of such a case with a primary EACC in a male which was managed by modified radical mastoidectomy.

## 2. Case Report

A 30-year-old male presented with complaints of left ear intermittent scanty discharge for 10 years and a decreased left hearing for 1 year.

On otoscopic examination, a foul smelling discharge was present along with an aural polyp occluding the EAC opening. Rinne was negative, and Weber was lateralized to the affected ear.

**Figure 1:**
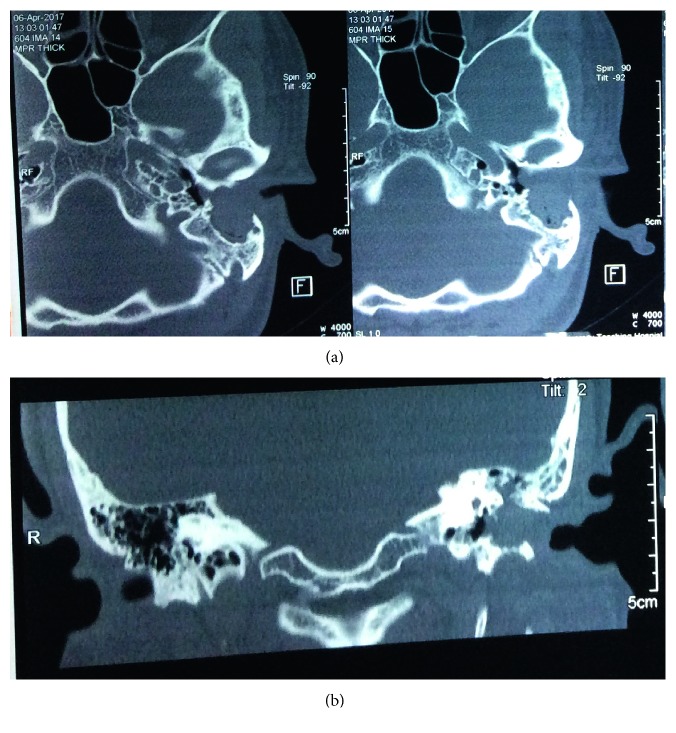
(a) High-resolution computed tomography (HRCT) temporal bone axial view showing homogeneous soft-tissue density filling the left EAC extending into the mastoid cavity destroying the posterior wall of EAC, focal destruction of anterior canal wall. Medial bowing of the tympanic membrane can also be seen. (b) High-resolution computed tomography (HRCT) temporal bone coronal view showing homogeneous soft-tissue density filling the left EAC extending superiorly and inferiorly with focal bony destruction.

High-resolution computed tomography (HRCT) showed homogeneous soft-tissue density, filling the left EAC, extending into the mastoid cavity, destroying the posterior wall of the EAC. Medial bowing of the tympanic membrane could also be seen. Focal bony destruction could be seen in inferior EAC (Figure 1).

**Figure 2:**
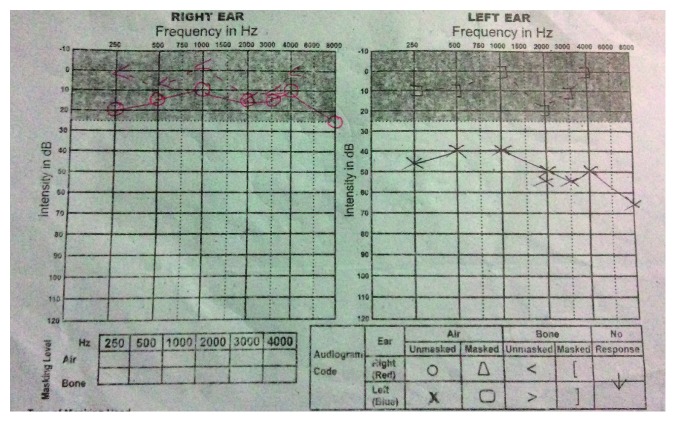
Pure tone audiometry. Right ear: normal with 13 dB; left ear: moderate conductive type hearing loss with 47 dB.

Pure tone audiometry showed a normal right ear hearing level of 13 dB pantonal and moderate conductive type of hearing loss with 47 dB in the left ear (Figure 2).

**Figure 3:**
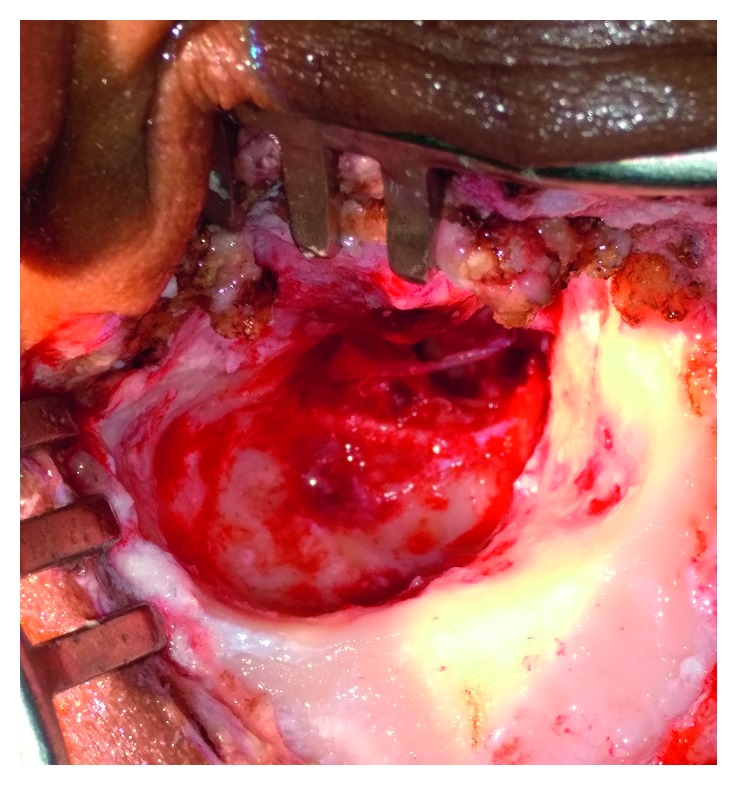
Huge cavity was formed by the disease with facial nerve dehiscent at horizontal, 2nd genu, and vertical portion with intact sheath.

The patient underwent modified radical mastoidectomy with graft only. The ear examination under the microscope revealed a retracted intact pars tensa and pars flaccida and a polyp arising from the facial ridge and the remnant posterior canal wall. The posterior canal wall was lowered up to the vertical part of the facial ridge by the disease. The cholesteatoma was present in the antrum, periantral, retrofacial, retrolabyrinthine, sinodural angle, tip cells, and around the sinus plate. Granulation tissue was present along the stapes. The facial nerve was dehiscent at the horizontal, 2nd genu, and vertical portion with an intact sheath (Figure 3). Malleus and incus were intact and mobile. However, the suprastructure of the stapes was absent, and the footplate was mobile after removing the overlying granulation tissue. Pars tensa and attic were normal. Temporalis fascia grafting was done.

The three-month follow-up examination showed a healthy cavity.

## 3. Discussion

Though cases of the EACC have been reported as early as 1850 by Toynbee [4] and 1893 by Scholefield [5], some authors speculate that they might have represented keratosis obturans due to similar characteristics [2]. Present definitions have been based upon the review by Piepergerdes et al. in 1980 [6] and histopathological study by Naiberg et al. in 1984 [7]. Persaud et al. reviewed the literature in attempt to define clearer distinctions. Their only conclusion was that there are still no reliable consistent symptoms or clinical signs that can differentiate between the two conditions; however, the most useful finding confirming an EACC is focal osteonecrosis or sequestration of bone lacking an epithelial covering [8].

Tos has classified EACC based on pathogenetic theories into (1) primary EACC, (2) secondary EACC, and (3) cholesteatoma associated with congenital atresia of the ear canal [9]. The aetiology of primary EACC is unknown [9]. Some have hypothesized that EACC is a reactive process due to a primary underlying osteitis [1, 6, 7]. Smoking and mechanical factors (use of Q-tips and hearing aid) may be predisposing factors [2]. Secondary EACC is related to a variety of different causes, such as postoperative, postinflammatory, postirradiatory, posttraumatic, or postinflammatory stenosis or atresia of the external auditory canal in descending order of frequency [2, 9, 10]. Dubach and Hausler have found two cases of EACC in patients with Langerhans cell histiocytosis [11].

There is no gender, age, or side preponderance. Though the most common presenting symptoms reported in the literature are otalgia and otorrhea, patients may present with external ear canal occlusion, hearing loss, itching, or even asymptomatic signs. Secondary cases are usually less extensive than primary or less prominent symptoms [2]. EACC is usually found in anterior, inferior, and posterior EAC and rarely superiorly or circumferential [1, 2, 6]. In our case, the main complaints of our patient were otorrhea and a decreased hearing. The disease was extensive with a destruction of the EAC circumferentially, exposing facial nerve, bowing of pars tensa touching the medial wall of the mesotympanum (Figure 3). Naim et al. have classified EACC into four stages: stage I with hyperplasia of the canal epithelium, stage II including periosteitis, stage III including a defective bony canal, and stage IV showing an erosion of adjacent anatomic structures [12]. According to which our case can be classified as a stage IV EACC.

Differential diagnosis includes keratosis obturans, postinflammatory medial canal fibrosis, malignant otitis externa, and neoplasms of the EAC. For better differentiation and to determine the true extent of the disease which might be inapparent at clinical examination, imaging is strongly recommended. Temporal bone CT shows EACC as a soft-tissue mass within the EAC, with adjacent bone erosion. Bone fragments may be present within the mass. The cholesteatoma may extend into adjacent structures [13].

Differentiating it from keratosis obturans, hearing loss due to EACC is mostly infrequent; the pain sensation is more dull and less acute than in keratosis obturans [4]. The lesions are more localized, and the tympanic membrane generally appears normal in contrast to keratosis obturans in which inflammation of the ear canal skin and tympanic membrane is seen [6, 8]. Focal skin disruption, osteonecrosis, and varying sequestration should favour a diagnosis of EACC [2]. In low number of cases, an invasion of adjacent structures has been seen more commonly in the mastoid, middle ear, temporomandibular joint, and less frequently in erosions of the facial nerve, tegmen, atticus, and antrum [2, 13]. More recently, immunohistochemical investigations have been introduced, reporting increased levels of various growth factors in EACC specimens [14].

Small lesions can be treated conservatively or by minimal procedures under local anesthesia, while larger lesions need proper surgery with removal of the cholesteatoma, burring off the affected bone areas, and grafting the defects with fascia. Sometimes even small lesions have to be treated aggressively if resistant to conservative management [1].

## 4. Conclusions

External auditory canal cholesteatoma (EACC) is a rare condition which can be easily diagnosed and differentiated radiologically from keratosis obturans and can be managed by modified radical mastoidectomy.
